# Mechanisms of recurrent haemoptysis after super-selective bronchial artery coil embolisation: a single-centre retrospective observational study

**DOI:** 10.1007/s00330-018-5637-2

**Published:** 2018-07-19

**Authors:** Misaki Ryuge, Masahiko Hara, Takanori Hiroe, Naoki Omachi, Shojiro Minomo, Kazushi Kitaguchi, Mihoko Youmoto, Norihiro Asakura, Yasushi Sakata, Hideo Ishikawa

**Affiliations:** 1 Hemoptysis and Pulmonary-Circulation Center, Kishiwada Eishinkai Hospital, Kishiwada, Japan; 2 Center for Community-based Healthcare Research and Education, Shimane University, Enya-cho 223-8, Zip code, Izumo, 693-8501 Japan; 3 Department of Biostatistics, Kyoto University Graduate School of Medicine, Kyoto, Japan; 4 Department of Internal Medicine, National Hospital Organization Kinki-chuo Chest Medical Center, Osaka, Japan; 5 Department of Cardiovascular Medicine, Osaka University Graduate School of Medicine, Suita, Japan

**Keywords:** Bronchial arteries, Haemoptysis, Recurrence, Observational study

## Abstract

**Objectives:**

In recognition of the significant impairment caused by haemoptysis on a patient’s quality of life, bronchial artery embolisation has been introduced worldwide as one of the first-line treatment options. Since little evidence is available on the mechanisms of recurrent haemoptysis after super-selective bronchial artery coil embolisation (ssBACE), the purpose of the present study is to evaluate these.

**Methods:**

We retrospectively evaluated the mechanisms of recurrent haemoptysis using both enhanced computed tomography and cineangiography following ssBACE by reviewing 299 haemoptysis-related arteries (HRAs) in 57 consecutive patients who underwent 2nd series ssBACE for the management of recurrent haemoptysis between April 2010 and December 2015.

**Results:**

Median age of patients was 69 (interquartile range 64–74) years, and 43.9% were men. This study revealed that (1) recanalisation was the most common mechanism (45.2%) followed by development of new HRA (38.5%), bridging collaterals (14.7%) and conventional collaterals (1.7%); (2) these trends could be modified in several situations such as with antiplatelet or anticoagulant medications; (3) relatively large-diameter HRAs were more likely to recanalise compared with small-diameter HRAs and (4) recurrent haemoptysis could be managed by 2nd series ssBACE with a procedural success rate of 97.7% without any major complications.

**Conclusions:**

Recanalisation was the most common mechanism of recurrent haemoptysis after ssBACE. Our results provide interventionists with indispensable insights.

**Key Points:**

*• Recanalisation was the most common mechanism of recurrent haemoptysis after super-selective bronchial artery coil embolisation, followed by development of new haemoptysis-related arteries*

*• These trends could be modified in several situations such as with antiplatelet or anticoagulant medications*

*• Recurrent haemoptysis could be managed by 2nd series super-selective bronchial artery coil embolisation with a procedural success rate of 97.7% without any major complications.*

## Introduction

Haemoptysis affects patients with a wide variety of respiratory diseases, such as bronchiectasis, non-tuberculous mycobacterium (NTM) pulmonary infection and pulmonary aspergillosis [1–6]. As a result of the significant negative impact of massive haemoptysis on a patient’s quality of life and risk of mortality, an interventional radiological approach known as bronchial artery embolisation (BAE) has been introduced and evaluated extensively [7–14]. In addition, interest has recently started to shift to long-term haemoptysis-free survival rather than short-term haemostatic rate, since short-term haemostasis can be achieved in the majority of cases owing to advancements in interventional devices and medical knowledge [9–17]. For example, BAE performed using polyvinyl alcohol (PVA) or *N*-butyl-2-cyanoacrylate (NBCA), or using a metallic coil, has been shown to produce much more favourable long-term outcomes compared to BAE performed using a gelatin sponge [14, 16]. The largest observational studies to date have shown that the estimated 1-year haemoptysis-free survival rates for BAE performed using PVA, NBCA and a metallic coil are 77%, 88% and 87%, respectively [14, 16]. However, little evidence is available on the mechanisms of recurrent haemoptysis after BAE using metallic coil [11–17]. The aim of the present study was to evaluate the mechanisms of recurrent haemoptysis using both enhanced-computed tomography (e-CT) and cineangiography after super-selective bronchial artery coil embolisation (ssBACE) by reviewing 299 haemoptysis-related arteries (HRAs) from 57 consecutive patients who underwent 2nd series ssBACE for the management of recurrent haemoptysis.

## Materials and methods

### Study patients and data collection

This single-centre retrospective study included 57 consecutive patients who underwent 2nd series ssBACE for the management of recurrent haemoptysis after 1st series elective ssBACE (*n* = 489) at our institution between April 2010 and December 2015 [16]. As previously reported, we defined haemoptysis and recurrent haemoptysis as airway bleeding with an estimated volume of greater than 20 cm^3^. In this study, ssBACE was indicated for patients with severe impairments to daily quality of life regardless of the amount of haemoptysis [16]. We collected data on all the variables shown in tables and figures retrospectively from the patient records. In the present study, underlying diseases included bronchiectasis, NTM pulmonary infection, pulmonary aspergillosis, pulmonary tuberculosis (Tb) sequelae and cryptogenic haemoptysis. Exacerbation of underlying diseases was evaluated by the consensus of two pulmonologists on the basis of CT findings. We defined exacerbation as being present when the number or extent of disease-related findings increased in the lung parenchyma on CT at 2nd series ssBACE compared with the findings at 1st series ssBACE. For example, exacerbation of bronchiectasis was determined if new enlargement or increase of dilated bronchus was present. Decision was withheld if the presence of blood significantly impaired accurate interpretation by masking lung parenchymal changes because of exacerbation of underlying diseases (*n* = 1). The study protocol complied with the standards outlined in the Declaration of Helsinki and was approved by the institutional ethical committee (approval number 2017-03). The requirement of written informed consent was waived because of the retrospective nature of the study. Several cases have previously been published as image-content sharing case reports in another journal [18].

### Standard ssBACE procedure and classification of mechanisms

**Fig. 1:**
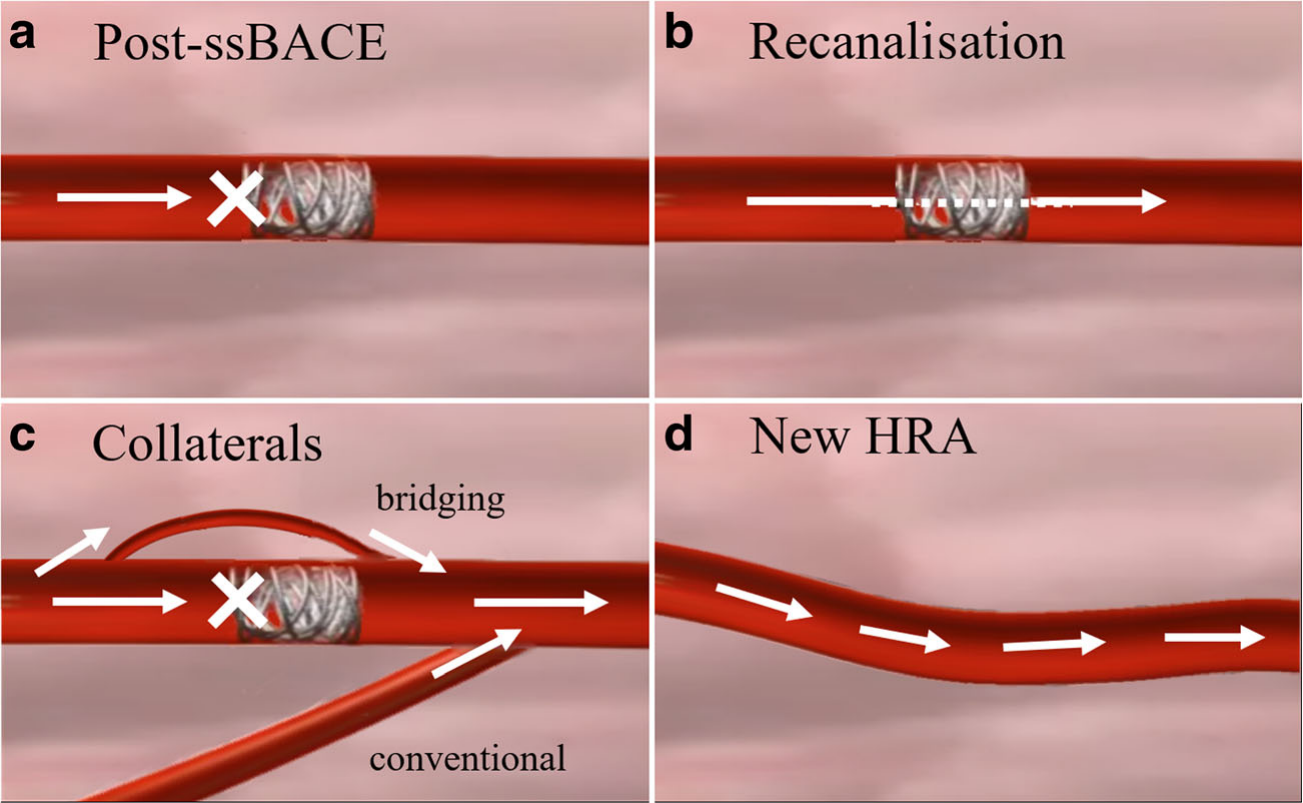
Schematic diagram of mechanisms of recurrent haemoptysis after ssBACE. White arrows indicate blood flow. HRA is successfully embolised after 1st series ssBACE (**a**). Four possible mechanisms of recurrent haemoptysis include recanalisation (**b**), bridging and conventional collaterals (**c**) and the development of new HRA (**d**). We defined conventional collaterals as when the distal part of an embolised HRA receives blood from other vessels rather than the proximal part of the embolised HRA. HRA haemoptysis-related artery, ssBACE super-selective bronchial artery coil embolisation

The standard ssBACE procedure and identification protocol for HRAs have previously been reported elsewhere and are summarised shortly as follows [16]. During 1st series ssBACE, HRAs identified using e-CT were embolised using three detachable or pushable platinum coils (IDC® or Interlock® Boston Scientific Japan, Target® Stryker Japan, Nester® Cook Japan, C-STOPPER® PIOLAX). The first coil deployment was performed for anchoring, followed by filling and finishing coil deployments [16]. Before the 2nd series ssBACE, all patients underwent e-CT angiography again to evaluate possible HRAs for procedural planning [5, 6, 9, 16, 19]. Primary signs of a possible HRA consisted of dilatation of the vessel compared to the normal size for that site, tortuosity of the vessels, direct shunting of vessels, aneurysmal formation, pleural adhesion, ground glass opacity suggesting inhaled blood, and new enhancement of the distal part of the HRA embolised at the 1st series ssBACE [1, 5, 6, 8, 9, 16]. Considering the normal arterial vessel size, we defined relatively large-diameter HRAs as those occurring in the bronchial, intercostal and internal thoracic arteries, and relatively small-diameter HRAs as those occurring in all other arteries. All possible HRAs identified using e-CT were super-selectively evaluated using arteriography with 5-Fr guiding catheter and 3-Fr microcatheter systems, with 0.014- or 0.016-inch guide wires, during the 2nd series ssBACE [16]. Aortography was not performed. Following the comprehensive evaluation of e-CT and cineangiography, we classified the mechanisms of recurrent haemoptysis into four categories (as shown in Fig. 1): (1) recanalisation, (2) conventional collaterals, (3) bridging collaterals and (4) new HRA. This last category included vessels that were overlooked (*n* = 4) or ones that could not be embolised (*n* = 3) during the 1st series ssBACE. We defined conventional collaterals as instances where the distal part of an embolised HRA is fed blood from vessels other than the proximal part of the embolised HRA. Conversely, we defined bridging collaterals as instances when the distal part of an embolised HRA is fed blood directly from the proximal part of the embolised HRA. Second series ssBACE was performed at the proximal part of previously embolised HRAs if recanalisation or bridging collaterals were present, whereas for newly developed HRAs or if conventional collaterals were present, 2nd series ssBACE was performed in the same manner as the 1st series ssBACE. Procedural success rate was calculated as the number of successfully embolised HRAs divided by the total number of target HRAs during the ssBACE procedure. The definition of major and minor complications was based on guidelines from the Society of Interventional Radiology Standards of Practice Committee [20].

### Statistical analysis

Our primary outcome of interest was the frequency of occurrence of each mechanism of recurrent haemoptysis. The incidence of each mechanism was counted on the basis of HRA (*n* = 299). Continuous variables were summarised using medians and interquartile ranges (quartiles 1 to 3), and categorical variables were summarised by percentages. Differences in the incidence of each mechanism between categories were evaluated using a Fisher’s exact test, and those among underlying diseases or vessel types were evaluated, focusing on whether recanalisation is more common as a cause of recurrent haemoptysis than any other cause. By contrast, differences in continuous variables were evaluated using a Kruskal–Wallis test. All statistical analyses were performed using Microsoft R open software (version 3.3.2).

## Results

Patients’ characteristics at the time of the 1st ssBACE are shown in Table 1. The median age of patients was 69 (64–74) years old, and 43.9% were men. The most common underlying disease of recurrent haemoptysis after ssBACE was bronchiectasis (38.6%), followed by NTM pulmonary infection (35.1%) and pulmonary aspergillosis (17.5%). The median time from the 1st ssBACE to recurrent haemoptysis was 218 (57–452) days, and there were significant differences among underlying diseases (*p* = 0.020): the shortest time was 7 (5–9) days for patients with cryptogenic haemoptysis, and the longest time was 354 (165–534) days for patients with NTM pulmonary infection. The number of HRAs was 5 (3–8) and 50% of patients showed exacerbation of underlying diseases at 2nd series ssBACE. The procedural success rate of 2nd series ssBACE was 97.7%, and no major and one minor complication of slight asymptomatic mediastinal haematoma due to wire perforation of an internal thoracic artery which did not need additional management were reported. Regarding the characteristics of HRAs, relatively large-diameter arteries, such as the bronchial (29.8%), intercostal (28.8%) and internal thoracic (13.0%) arteries, were the most common sites of HRAs during the 2nd series ssBACE.

**Table 1:** Patient characteristics

Parameters	Total (*n* = 57)
Baseline data at 1st ssBACE series	
Age (years)	69 (64–74)
Male	43.9
Body mass index (kg/m^2^)	19.3 (17.6–21.5)
Current smoker	3.5
Underlying disease	
Bronchiectasis	38.6
NTM pulmonary infection	35.1
Pulmonary aspergillosis	17.5
Pulmonary Tb sequelae	5.3
Cryptogenic haemoptysis	3.5
Creatinine (mg/dL)	0.64 (0.53–0.73)
Number of HRA	6 (4–8)
Procedural success rate of 1st series ssBACE	99.1
2nd ssBACE series	
Recurrent haemoptysis from 1st series (days)	218 (57–452)
Bronchiectasis	185 (36–319)
NTM pulmonary infection	354 (165–534)
Pulmonary aspergillosis	59 (37–141)
Pulmonary Tb sequelae	354 (286–459)
Cryptogenic haemoptysis	7 (5–9)
	Kruskal–Wallis *p* = 0.020
Emergent ssBACE	12.3
On anticoagulant medication	8.8
On antiplatelet medication	7.0
Exacerbation of baseline disease on CT	50.0 *
Number of HRA	5 (3–8)
Recanalisation	80.7
New HRA	75.4
Bridging collateral	47.4
Conventional collateral	7.0
Procedural success rate of 2nd series ssBACE	97.7
Major complications	0.0
Minor complications	1.8

Continuous variables were summarised using medians and interquartile ranges (quartiles 1 to 3), and categorical variables were summarised by percentages

*CT* computed tomography, *HRA* haemoptysis-related artery, *NTM* non-tuberculous mycobacterium, *ssBACE* super-selective bronchial artery coil embolisation, *Tb* tuberculosis

*Could not be determined in one patient (*n* = 56 for this outcome)

**Fig. 2:**
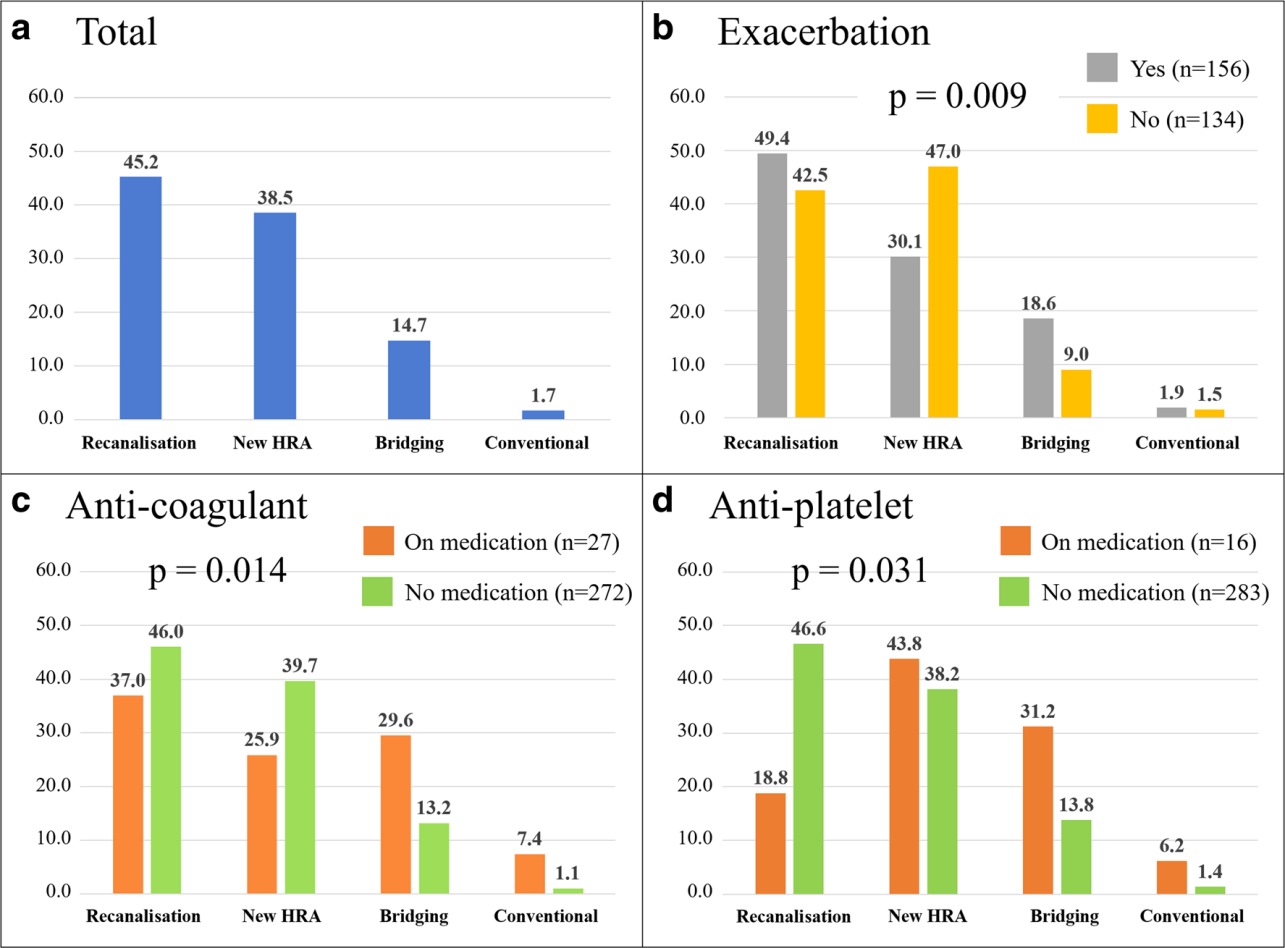
Incidence of each mechanism of recurrent haemoptysis after ssBACE. *n* in parentheses indicates the number of HRAs. HRA haemoptysis-related artery, ssBACE super-selective bronchial artery coil embolisation

**Fig. 3:**
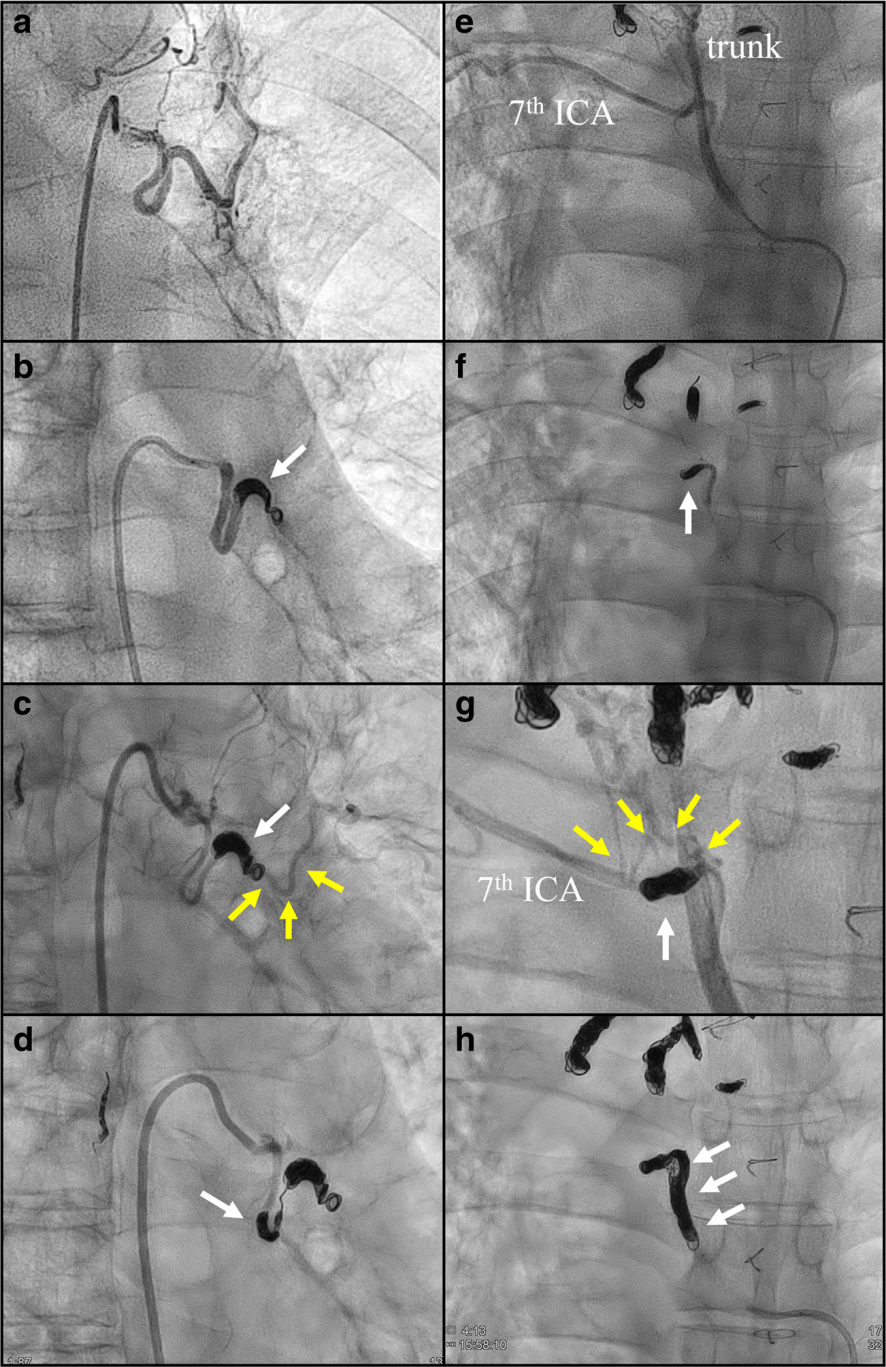
Representative cases of recanalisation and bridging collateral after ssBACE. Representative cineangiography findings of recanalisation (**a**–**d**) and bridging collateral (**e**–**h**) after 1st series ssBACE. Cineangiography revealed a dilated tortuous left bronchial (**a**) and right 7th ICA (**e**) with a bronchopulmonary shunt, which were embolised using metallic coil deployment during 1st series ssBACE (**b** and **f**; white arrows). For the treatment of recurrent haemoptysis, the patient underwent 2nd series ssBACE where cineangiography demonstrated a recanalisation (**c**; yellow arrows) or bridging collateral (**g**; yellow arrows) of the first embolised part of the HRA. Second series ssBACE was performed successfully at the proximal site of these HRAs (**d** and **h**; white arrows). ICA intercostal artery, HRA haemoptysis-related artery, ssBACE super-selective bronchial artery coil embolisation

The mechanisms of recurrent haemoptysis are shown in Fig. 2 and Table 2. In general, recanalisation (45.2%) of HRAs was the most common cause in patients with recurrent haemoptysis, followed by new HRA (38.5%), bridging collaterals (14.7%) and conventional collaterals (1.7%) (Fig. 2A). We have presented representative cases with recanalisation and bridging collaterals in Fig. 3 for reference. In a retrospective review of all new HRAs, four could be identified using e-CT evaluation at the 1st series ssBACE. The new HRA category includes the vessels that were overlooked (*n* = 4) or ones that could not be embolised (*n* = 3) at the time of the 1st series ssBACE. Several subsets of patients, such as those with no exacerbation of underlying disease (Fig. 2B) and those receiving an anticoagulant (Fig. 2C) or antiplatelet agent (Fig. 2D), showed statistically significant different trends.

**Table 2:** Mechanisms of recurrent haemoptysis based on HRA

	Recanalisation (*n* = 135)	New HRA (*n* = 115)	Bridging collateral (*n* = 44)	Conventional collateral (*n* = 5)
Baseline disease				
Bronchiectasis	50.5	35.5	11.2	2.8
NTM pulmonary infection	43.3	39.4	16.3	1.0
Pulmonary aspergillosis	41.9	38.7	17.7	1.6
Pulmonary Tb sequelae	55.6	22.2	22.2	0.0
Cryptogenic haemoptysis	0.0	100.0	0.0	0.0
	*p* = 0.046			
Target HRA				
Bronchial	58.4	23.6	18.0	0.0
Intercostal	43.0	39.5	14.0	3.5
Internal thoracic	51.3	25.6	23.1	0.0
Inferior phrenic	37.5	54.2	8.3	0.0
Supreme intercostal	31.2	50.0	18.8	0.0
Lateral thoracic	46.2	53.8	0.0	0.0
Thoracoacromial	36.4	45.5	0.0	18.2
Others	9.5	81.0	9.5	0.0
Pulmonary ligament	0.0	87.5	12.5	0.0
Inferior thyroid	20.0	80.0	0.0	0.0
Dorsal scapular	0.0	100.0	0.0	0.0
Thoracodorsal	50.0	50.0	0.0	0.0
Thyroid carotid	0.0	100.0	0.0	0.0
Superior thoracic	0.0	0.0	100.0	0.0
Pulmonary	0.0	100.0	0.0	0.0
	*p* = 0.001			

Categorical variables were summarised by percentages

*HRA* haemoptysis related artery, *NTM* non-tuberculous mycobacterium, *ssBACE* super-selective bronchial artery coil embolisation, *Tb* tuberculosis

The fact that relatively large-diameter arteries were the most common sites of HRAs is reflected by the general trend of mechanisms contributing to recurrent haemoptysis. Conversely, the most common mechanism of recurrent haemoptysis in relatively small-diameter arteries, such as the inferior phrenic, supreme intercostal, lateral thoracic, thoracoacromial or other arteries, was the development of new HRAs rather than recanalisation (*p* < 0.001; Table 2).

## Discussion

In the present study, we evaluated the mechanisms of recurrent haemoptysis after ssBACE by reviewing 299 HRAs from 57 patients. The main findings were that (1) recanalisation (45.2%) was the most common mechanism followed by the development of new HRAs (38.5%); (2) these trends differed according to whether patients were on antiplatelet or anticoagulant medications; (3) relatively large-diameter arteries were the most common sites of HRAs, and tended to recanalise more often compared to relatively small-diameter arteries and (4) recurrent haemoptysis could be managed by 2nd series ssBACE with a procedural success rate of 97.7% without any major complications. Since our study is the first to evaluate the mechanisms of recurrent haemoptysis after ssBACE, we believe that our results provide interventionists with indispensable insights for the management of haemoptysis. Further, the findings of this study will significantly improve prognosis and quality of life in patients who suffer from haemoptysis.

### Mechanism of recurrent haemoptysis

The most important finding of the present study concerns the mechanisms of recurrent haemoptysis after ssBACE. Recanalisation and the development of new HRAs caused more that 80% of the HRAs at the 2nd series ssBACE. In contrast to PVA and NBCA, which serve as bloodstream-dependent linear liquid embolisation materials, ssBACE provides patients with controlled pinpoint embolisation of target HRAs [10–17]. In light of this, we speculate that the most common mechanism of recurrent haemoptysis, namely recanalisation, may occur more frequently during ssBACE compared to BAE performed with PVA or NBCA. However, the mechanisms of recurrent haemoptysis following BAE using PVA or NBCA have yet to be investigated [10–17]. We also demonstrated that the development of new HRAs was the second most common cause of recurrent haemoptysis in this study, in which 108 (93.9%) were new development of HRA. Even though haemoptysis recurred in an unexpectedly short time period in two patients with cryptogenic haemoptysis (7 [5–9] days; Table 1), this may have been due to a rapid change of bloodstream distribution after the 1st series ssBACE because we could not identify these HRAs in a retrospective review of e-CT or cineangiography at 1st series ssBACE.

### Different mechanisms in specific situations

We also revealed that the mechanism of recurrent haemoptysis was significantly different among several subsets of patients. For example, new HRA was the most common mechanism for patients without exacerbation of underlying diseases, as well as in patients receiving an antiplatelet agent. These findings suggest that recurrent haemoptysis could potentially be controlled in certain cases using medication, but the clinical application of this management strategy requires further evaluations. In a subgroup analysis, the most common mechanism was different between large- and small-diameter HRAs. It is understandable that ssBACE of relatively large-diameter vessels could lead to low-density-coil deployment and result in a high incidence of recanalisation. To avoid this kind of recanalisation, or to reduce the incidence of recurrent haemoptysis, we speculate that a high-packing-density-coil deployment manoeuvre, using a hydrogel-polymer-coated platinum coil or a high thrombogenic coil, might be pertinent issues in this field. The efficacy of this manoeuvre has already been shown in neurovascular and gastrointestinal areas, as well as in the treatment of pulmonary arteriovenous malformations [21–23].

### Clinical implication

Our study has important practical clinical implications. First, one of the most effective strategies towards improving outcomes in patients with haemoptysis would be to reduce the number of recanalisation events after ssBACE, because we have shown that this is the most common mechanism of recurrent haemoptysis. The evaluation of recently introduced hydrogel-polymer-coated platinum coils is anticipated in future studies [21–23]. Second, we revealed that 2nd series ssBACE can be performed with a 97.7% procedural success rate even if the proximal part of HRAs was embolised during 1st series ssBACE. In this regard, it is important to remind that 1st series ssBACE should be performed saving some space proximally for additional coil deployment in possible future recanalisation risk. Considering that extreme caution should be exercised when using bloodstream-dependent liquid embolisation materials, owing to the risk of misembolisation particularly in the spinal branches [24, 25], the repeatability of controlled pinpoint embolisation of ssBACE can be a strength for safe and effective BAE procedures in theory [15–17].

### Study limitations

Our study was retrospective and observational in nature, and it was conducted at a single site. In addition, we analysed data with the assumption that each HRA measurement would be independent because our interest lay in the phenomenon that occurred in each HRA. Thus, statistical significance might have been overestimated compared with patient-based analysis. However, this study examined the largest number of recurrent haemoptysis patients to date and provides the first comprehensive evaluation of the mechanisms of recurrent haemoptysis after ssBACE. These strengths, we feel, outweigh the limitations of the present study.

## Conclusions

Recanalisation was the most common mechanism of recurrent haemoptysis after ssBACE. Our results provide interventionists with indispensable insights for the management of haemoptysis.

## Abbreviations

BAE: Bronchial artery embolisation
e-CT: Enhanced-computed tomography
HRAs: Haemoptysis-related arteries
NBCA: *N*-butyl-2-cyanoacrylate
NTM: Non-tuberculous mycobacterium
PVA: Polyvinyl alcohol
ssBACE: Super-selective bronchial artery coil embolisation
